# Impact of opioid analgesics on the efficacy of immune checkpoint inhibitors in a lung cancer population

**DOI:** 10.1186/s12890-022-02210-9

**Published:** 2022-11-21

**Authors:** Xiaoyuan Yu, Li Zhao, Bin Song

**Affiliations:** 1 First Hospital of Shanxi Medical University, Taiyuan, China; 2 Third Hospital of Shanxi Medical University, Shanxi Bethune Hospital, Shanxi Academy of Medical Sciences, Tongji Shanxi Hospital, Taiyuan, 030032 China

**Keywords:** Lung cancer, Immune checkpoint inhibitors, Drug resistance, Opioid analgesics, Gut microbiota

## Abstract

**Objective:**

A retrospective clinical study was conducted to compare the prognosis between the opioid analgesic (OA) treated and OA-untreated groups and to evaluate the effect of opioid analgesics on the efficacy of immune checkpoint inhibitors (ICIs) in the treatment of advanced lung cancer patients. In addition, a subgroup analysis of the clinical characteristics of the enrolled patients was performed to explore possible influencing factors.

**Methods:**

This study reviewed the medical records of eligible patients who received ICIs at our institution. The clinicopathological features and clinical outcomes were compared. Also, the use of OA was collected. Patient survival, the incidence of immune-related adverse events (irAEs), and other baseline variables were examined in both cohorts according to whether OA was used.

**Results:**

A total of 132 patients were included in the study. Of them, 39 (29.5%) were in the OA-treated group. No significant differences in baseline characteristics were observed between the OA-treated and untreated groups. The combined application of OA treatment significantly shortened progression-free survival (PFS) (*P* < 0.001) and overall survival (OS) (*P* = 0.002). However, both groups experienced similar incidences and gradations of irAEs. According to multivariate analysis, OA treatment resulted in significantly worse PFS (HR = 4.994, 95% CI 3.217–7.753, *P* < 0.001) and OS (HR = 3.618, 95% CI 2.030–6.240, *P* < 0.001).

**Conclusions:**

Clinical outcomes of ICIs were significantly diminished in a cohort of Chinese patients with advanced lung cancer receiving OA therapy.

## Introduction

With the further elucidation of cancer physiological mechanisms, cancer-immune system interactions play an essential role in cancer development. Immune checkpoint inhibitors (ICIs) have been recognized as one of the most effective approaches against tumors due to their pioneering access to specific anti-cancer mechanisms [1]. In clinical practice, individual differences in the efficacy of ICIs are evident, with objective response rates of only about 20% in unselected populations [2]. Prediction of the effectiveness of ICIs still plagues clinicians' decision-making. Exploring the interaction of different drugs helps to screen sensitive populations, improve the efficacy of ICIs, and improve the prognosis of patients.

Studies show that gut microbiota loss and altered composition can negatively affect systemic immune responses by altering cytokine production and T-cell function, and NK cell activity, thereby diminishing the therapeutic efficacy of ICIs [3, 4]. The composition and number of gut microbiota are disturbed by a variety of factors, including gender, age, diet, history of smoking, and history of medication use. Opioid analgesics ( OA) are frequently and widely used in patients with malignant tumors. OA has a significant impact on intestinal flora composition and human immune system, which are considered to be important factors affecting the efficacy of ICIs [5, 6]. In some small sample studies, a debilitating effect of OA can be observed [7]. However, Chinese patients were not included in this study, which poses a challenge to the comprehensiveness of the relevant studies. To fully understand the relationship between OA treatment and ICIs efficacy, more data from different patient cohorts is required.

This study aimed to conduct a retrospective cohort study by collecting clinical data before ICIs treatment, and to examine prognostic impact after treatment, using OA as an exposure factor.

## Materials and methods

### Patients

Patients with advanced lung cancer attending the First Hospital of Shanxi Medical University and Shanxi Bethune Hospital from January 2019 to October 2021 were collected and screened. The patients all received ICIs, either monotherapy or in combination with chemotherapy and antiangiogenic therapy. Detailed patient information was collected using electronic medical records, mainly including age, gender, smoking history, family history, tissue type, primary tumor site, surgical history, performance status (PS), treatment route, treatment regimen, death date, and last follow-up visit. Tumor response was assessed according to the Immune-related Response Evaluation Criteria In Solid Tumors (iRECIST). Evaluation of adverse events (AEs) followed the Common Terminology Criteria for Adverse Events (version 4.0). Surgical history refers to surgery for the primary lung cancer lesion and was used to determine whether the patient was a postoperative recurrence. History of OA treatment refers to treatment with OA prescribed by the patient as needed and determined by the clinician. OA-treated patients were those who had received OA within one month before their first application of ICIs or during the treatment. This study has been approved by an institutional ethics review (No.SBQKL-2022–111).

### Statistical analysis

Using Fisher's exact test or chi-square test, we analyzed the relationship between clinicopathological variables. PFS is defined as the period from the date of initiation of ICIs treatment to the date of disease progression or death, whichever occurred first. OS was determined from the time of ICIs treatment initiation to the time of death from any cause or the last follow-up visit. The PFS and OS curves were determined using the Kaplan–Meier method and compared using the Log-Rank test and Breslow test. Univariate and multifactorial analyses were performed using the COX proportional risk model, and hazard ratios (HR) and 95% confidence intervals (CI) were calculated. We selected variables with *P* < 0.1 from the univariate analysis for the multivariate analysis. SPSS statistical software (version 26.0, IBM Corporation) was used for the statistical analysis. Two-sided *P*-values were calculated, and *P* < 0.05 was considered significant.

## Results

### Patient characteristics

In the First Hospital of Shanxi Medical University and Shanxi Bethune Hospital, 132 patients were included. There were 54 cases of squamous cell carcinoma (40.9%), 49 cases of adenocarcinoma (37.1%) and 29 cases of small cell lung cancer (22.0%). Patients were divided into two groups according to the grouping criteria: 39 patients (29.5%) in the OA-treated group and 93 patients (70.5%) in the OA-untreated group. At the time of data cut-off (March 2022), progressive disease (PD) events as defined by iRECIST were observed in 101 (76.5%) patients in the PFS analysis and 56 (42.4%) patients died in the OS analysis. Baseline assessments were performed for both cohorts and no statistically significant differences were seen. In Table 1, we summarize the clinicopathologic characteristics of all the patients included.

**Table 1:** Demographic data and clinical characteristics

Variable	Total (*N* = 132)	OA-Treated (*n* = 39)	OA-Untreated (*n* = 93)	*P* Value
Age, yr, No (%)				
Median, Range	63(30–88)	62(38–81)	64(30–88)	
≤ 65	84(63.6)	26(66.7)	58(62.4)	0.639
> 65	48(36.4)	13(33.3)	35(37.6)	
Gender, No (%)				
Female	35(26.5)	12(30.8)	23(24.8)	0.473
Male	97(73.5)	27(69.2)	70(75.2)	
Smoking, No (%)				
No	60(45.5)	21(53.8)	39(41.9)	0.210
Yes	78(54.5)	18(46.2)	54(58.1)	
ECOG PS, No (%)				
0	55(41.7)	20(51.3)	35(37.6)	
1	22(16.7)	6(15.4)	16(17.2)	0.293
2	47(35.6)	10(25.6)	37(39.8)	
3	8(6.0)	3(7.7)	5(5.4)	
Histology, No (%)				
Squamous	54(40.9)	16(41.0)	38(40.9)	0.208
Adenocarcinoma	49(37.1)	11(28.2)	38(40.9)	
Small cell	29(22.0)	12(30.8)	17(18.2)	
Neoplasm staging, No (%)				
III	9(6..8)	2(5.1)	7(7.5)	0.285
IV	106(80.3)	33(84.7)	73(78.5)	
Recurrent	17(12.9)	4(10.2)	13(14.0)	
Family History, No (%)				
Yes	20(15.2)	8(20.5)	12(12.9)	0.266
No	112(84.8)	31(79.5)	81(87.1)	
No..of treatment line. No (%)				
1	40(30.3)	7(17.9)	33(35.5)	0.293
2	77(58.3)	28(71.8)	49(52.7)	
3	12(9.1)	3(7.7)	9(9.7)	
4	3(2.3)	1(2.6)	2(2.2)	
Recurrence after operation, No (%)				
Yes	17(12.9)	4(10.3)	13(14.0)	0.560
No	115(87.1)	35(89.7)	80(86.0)	
Treatment regimen, No (%)				
ICIs monotherapy	26(19.7)	10(25.6)	16(17.2)	0.545
ICIs + chemo	91(68.9)	26(66.7)	65(69.9)	
ICIs + anti-EGFR	2(1.5)	0(0.00)	2(2.2)	
ICIs + anti-angiogenesis	13(9.8)	3(7.7)	10(20.8)	

### Correlation between OA use and outcomes

**Fig. 1:**
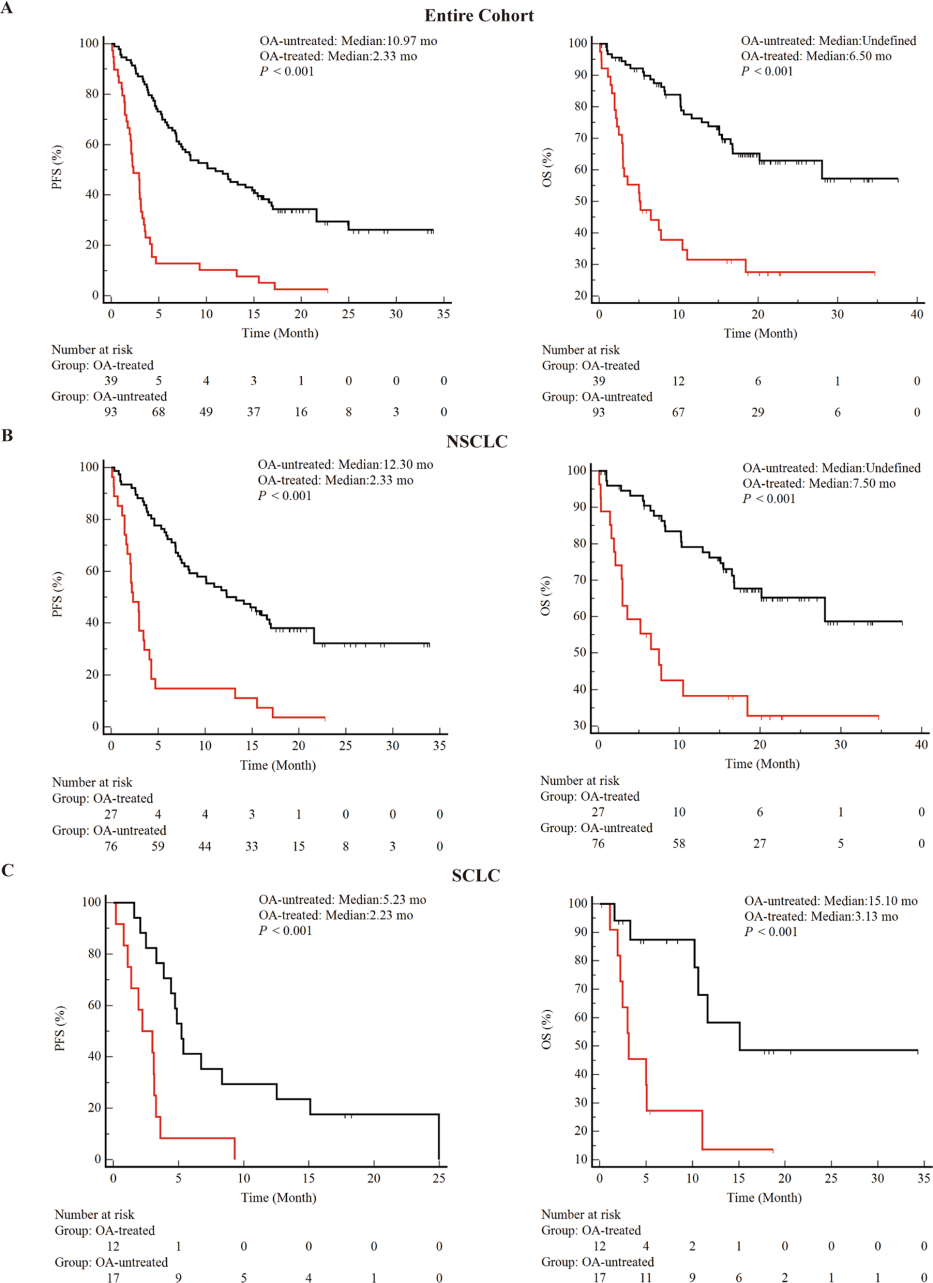
Correlation between combined histologic types and clinical outcomes during ICIs treatment. **A**: PFS and OS for the entire cohort. **B**: PFS and OS for patients with NSCLC (**C**): PFS and OS for patients with SCLC. abbreviations. OA, opioid analgesic; NSCLC, non-small cell lung cancer; SCLC, small cell lung cancer; mo, months; PFS, progression-free survival; OS, overall survival

**Fig. 2:**
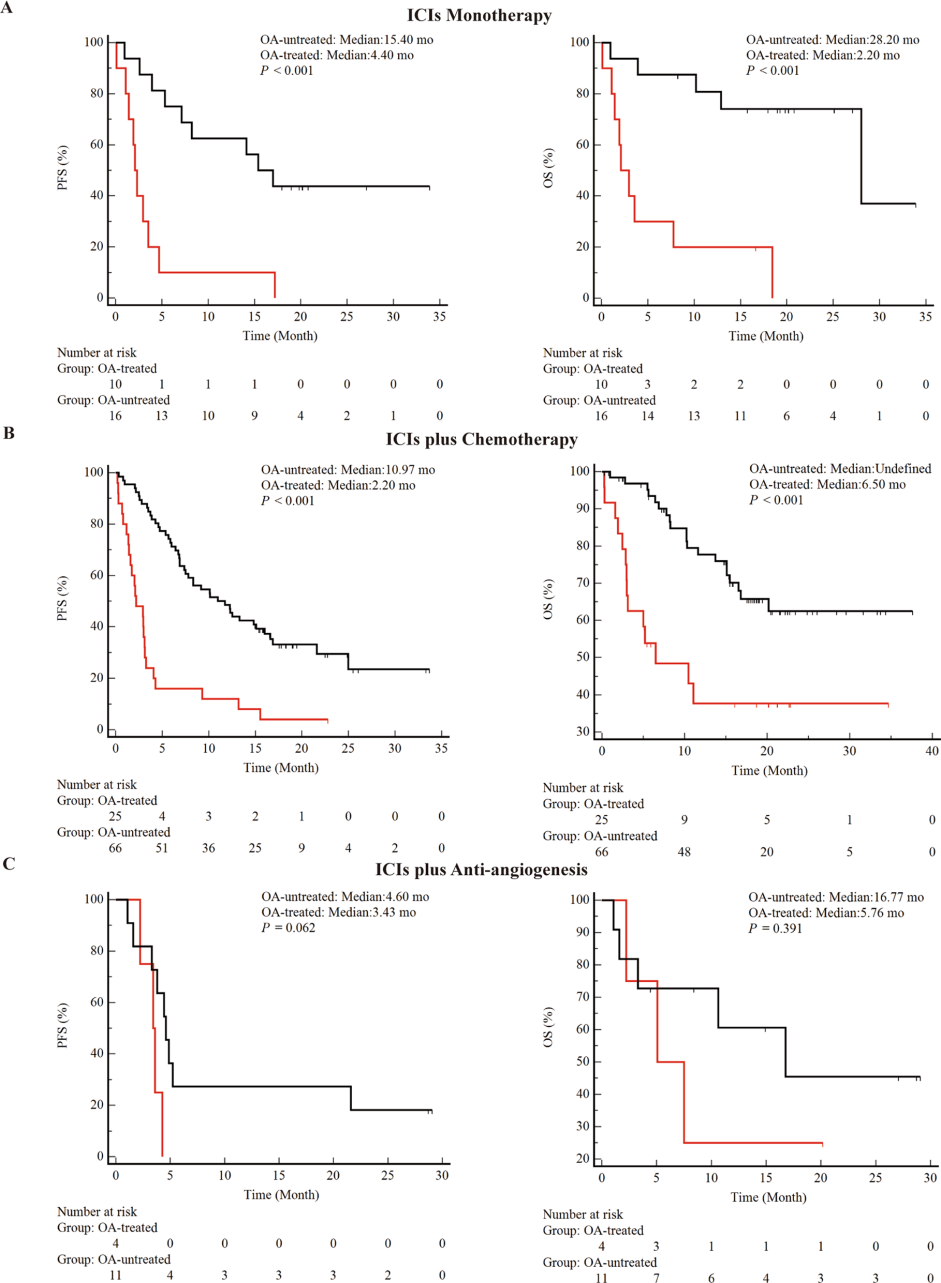
The correlations of concomitant OA treatment and clinical outcomes during ICIs therapies. **A**: PFS and OS for patients receiving ICIs monotherapy. **B**: PFS and OS for patients treated with ICIs plus chemotherapy. **C**: PFS and OS for patients treated with ICIs plus anti-angiogenesis. Abbreviations: OA, opioid analgesics; mo, months; PFS, progression-free survival; OS, overall survival

**Fig. 3:**
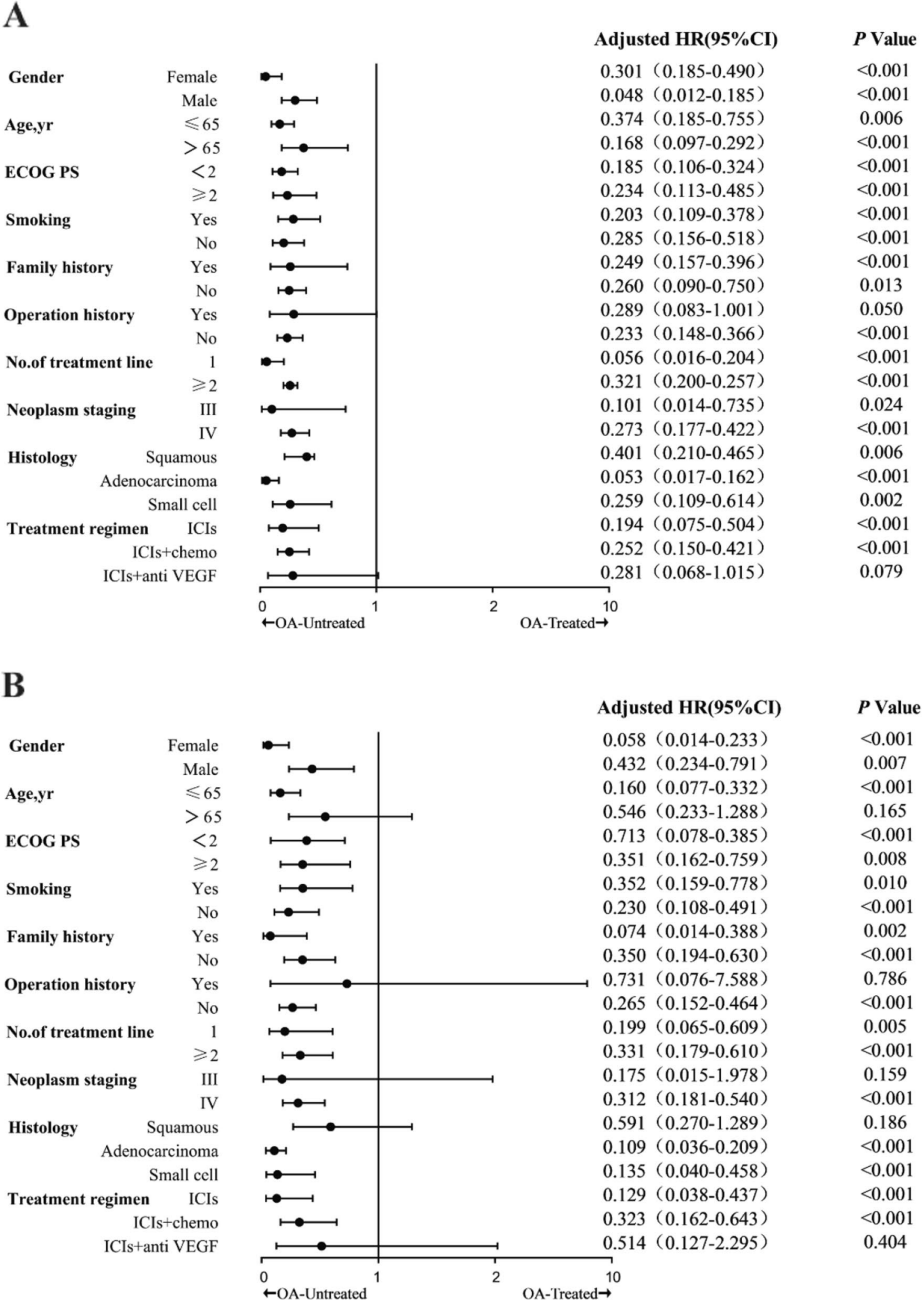
Forest plot of subgroup analysis by baseline characteristics for PFS (**A**) and OS (**B**) in all the included patients. Abbreviations: PFS, progression-free survival; OS, overall survival; OA, opioid analgesics; PS, performance status

As a first step, this study determined whether OA treatment affected clinical outcomes during ICIs treatment (Figs. 1 and 2). In the entire cohort, combined OA was associated with shorter PFS (median 2.33 vs 10.97 months, *P* < 0.001) and OS (median 5.50 vs not determined, *P* < 0.001) (Fig. 1A). In addition, the study performed inter cohort comparisons across tissue types (Fig. 1B and 1C). Finally, the study was compared between cohorts with different treatment regimens. Attenuation of PFS and OS of ICIs by OA was observed in both the ICIs monotherapy group (Fig. 2A) and the ICIs combined with the chemotherapy group (Fig. 2B). However, immune combined with anti-vascular therapy (Fig. 2C) did not show differences in the two cohorts. Subgroup analysis showed a negative effect of OA on survival during treatment with ICIs, with a decrease in PFS and OS in almost all subgroups discussed (Fig. 3).

### OA use and toxicity

Both cohorts experienced irAEs to vary degrees during treatment with ICIs (Table 2). The most common and common irAEs ( incidence ≥ 1%) in the cohort were neutropenia (9 cases, 68%), pulmonary toxicity (7 cases, 5.3%), thyroiditis (6 cases, 4.5%), skin toxicity (5 cases, 3.8%), and abnormal liver function (3 cases, 2.3%). Most patients experienced mild grade 1 or 2 irAEs, and no patient discontinued treatment because of irAEs. The irAEs were grade 3 in 8 cases (7 neutropenia, 1 skin toxicity), and grade 4 or higher irAEs did not occur. Neither the incidence (*P* = 0.648) nor the severity (*P* = 0.412) of irAEs were significantly different between the OA-treated and OA-untreated groups.

**Table 2:** The incidence of irAEs

Variable	OA-Treated (*n* = 39)	OA-Untreated (*n* = 93)	*P* Value
Any irAEs, No (%)			
Yes	9(23.0)	25(26.9)	0.648
No	30(77.0)	68(73.1)	
Highest grade of irAEs, No (%)			
1–2	8(88.9)	19(76)	0.412
> 2	1(11.1)	6(23)	

### Univariate and multivariate analysis

These are the results of the univariate and multifactorial analyses (Table 3 and 4). Univariate analysis revealed that PFS (HR = 0.241, 95% CI 0.158–0.368, *P* < 0.001) and OS (HR = 0.279, 95% CI 0.164–0.475, *P* < 0.001) were significantly shorter in patients treated with concomitant OA than in those who did not receive OA. PFS and OS were also worse in patients with ECOG PS ≥ 2. Patients diagnosed with stage IV lung cancer had a significantly shorter PFS and OS. In addition, patients diagnosed with small cell lung cancer and those without ICI as first-line therapy had worse PFS. However, pathological staging and the number of lines treated did not show significant differences in OS. In addition to these significant factors, the variables identified in the univariate analysis at *P* < 0.1 were included in the multifactorial analysis of PFS and OS. In the multifactorial analysis, the type of pathology was a risk factor for PFS. Following adjustment for other confounders, concomitant OA therapy was a significant independent prognostic factor for PFS (HR = 4.994, 95% CI 3.217–7.753, *P* < 0.001) and OS (HR = 3.618, 95% CI 2.030–6.240, *P* < 0.001).

**Table 3:** Univariate and Multivariate Analyses of Clinical Parameters on PFS

Factor	Univariate Analysis		Multivariate Analysis	
	HR (95% CI)	*P* Value	HR (95% CI)	*P* Value
Age, year (< 65 vs. ≥ 65)	1.217 (0.813, 1.821)	0.340	——	——
Gender (female vs. male)	1.077 (0.689, 1.683)	0.745	——	——
Smoking (never vs. smoker)	0.685 (0.463, 1.102)	0.058	——	——
Family History(yes vs no)	1.012 (0.584, 1.703)	0.966	——	——
Family History (yes vs no)	1.858 (1.251, 2.760)	0.002	2.719(1.786, 4.159)	< 0.001
ECOG PS (< 2 vs ≥ 2)	1.323 (1.105,1.725)	0.038	1.509(1.160, 1.946)	0.002
Histology (NSCLC vs SCLC)	2.348 (1.139, 4.840)	0.021	2.713(1.035, 4.563)	0.040
Neoplasm staging(III vs IV)	1.738 (1.111, 2.720)	0.015	1.238(0.770, 1.992)	0.378
No. of treatment line (1 vs ≥ 2)	0.828 (0.453, 1.514)	0.539	——	——
Treatment regimen (combination vs. monotherapy)	1.181 (0.817,1.708)	0.377	——	——
OA treated (yes vs no)	4.141 (2.715, 6.317)	< 0.001	4.994(3.217, 7.753)	< 0.001

**Table 4:** Univariate and Multivariate Analyses of Clinical Parameters on OS

Factor	Univariate Analysis		Multivariate Analysis	
	HR (95% CI)	*P* Value	HR (95% CI)	*P* Value
Age, year (< 65 vs. ≥ 65)	1.542 (0.910, 2.612)	0.107	——	——
Gender (female vs. male)	0.799 (0.422 1.515)	0.492	——	——
Smoking (never vs. smoker)	0.655 (0.387, 1.108)	0.058	0.814(0.477, 1.387)	0.449
Family History (yes vs no)	1.150 (0.563, 2.348)	0.701	——	——
ECOG PS (< 2 vs ≥ 2)	1.969 (1.164, 3.331)	0.011	2.364(1.392, 4.017)	0.001
Histology (NSCLC vs SCLC)	1.054 (0.742, 1.497)	0.769	——	——
Neoplasm staging(III vs IV)	3.436 (1.072, 11.006)	0.038	2.834(0.873, 9.198)	0.083
No. of treatment line (1 vs ≥ 2)	1.637 (0.893, 3.000)	0.111	——	——
Recurrence after operation(yes vs no)	0.534 (0.213, 1.340)	0.182	——	——
Treatment regimen (combination vs. monotherapy)	0.928 (0.567, 1.517)	0.766	——	——
OA treated (yes vs no)	3.582 (2.103, 6.102)	< 0.001	3.618(2.030, 6.240)	< 0.001

## Discussion

With the introduction of ICIs into the clinic brought a fundamental change in the treatment paradigm for lung cancer. Frontier studies have shown that loss of gut microbiota diversity and altered composition can diminish the therapeutic efficacy of ICIs [8, 9]. Wargo JA, et al. [10] analyzed the composition and abundance of fecal bacteria in patients effective on anti-PD-1 therapy and later observed that the gut microbiota may be an important factor influencing anti-PD-1 therapy. In-depth studies on the mechanisms of the gut microbiota-immune system demonstrated that Fecal bacilli upregulate CD8^+^ T cells and antigen-presenting molecules, and therefore, the flora itself may have an antitumor effect [11].

OA has been shown to affect the diversity and composition of the gut microbiota [5, 12]. The impact of OA use on ICIs may be an area of research that needs to be focused on [7]. In the study of Impact of concomitant drugs, it can be observed that the application of OA is negatively correlated with the efficacy of ICIs [13]. However, in such study, Chinese patients were not included and the relationship between OA and ICIs was not specifically discussed, which challenges the comprehensiveness of relevant research conclusions. Our research has made up for the above defects.

Relevant studies have shown that pain symptoms seriously reduce the quality of life of cancer patients and are considered to be one of the risk factors for the prognosis of patients [14, 15], which may lead to selection bias in this study and affect the reliability of the conclusions. However, Xie et al. [16] observed in a prospective study of 983 patients with advanced cancer in China that perfect analgesia can significantly prolong the total survival time of patients with advanced cancer, so that they can obtain the same total survival time as patients without pain. Similar conclusions have also been reported [17, 18]. In order to avoid bias, a total of 132 patients with advanced lung cancer were enrolled in this study. At the same time, medical records were consulted, and patients with improved analgesia after OA treatment were selected as the intervention group and patients without pain as the control group.

To avoid bias between different tissue types and treatment regimens, we analyzed patients according to their different treatment regimens. A history of OA treatment was observed to be associated with reduced efficacy of ICIs in all groups. This regimen was not analyzed because only 2 patients (only in the non-OA treated group) were enrolled in the anti-EGFR combination ICIs. Existing studies have demonstrated possible signaling crosstalk between EGFR and PD-L1 [19], which may interfere with the efficacy of ICIs, and further expansion of the cohort may help to identify new contraindications to the combination. In the subgroup analysis of included patients, concomitant OA showed a long-lasting negative impact on survival during treatment with ICIs, with reductions in PFS and OS observed in almost every subgroup discussed. This suggests that OA, as a drug that can interfere with the composition of the gut microbiota, may have a long-lasting and profound negative impact on the immune function of the population under discussion.

In our study, the use of ICIs in first line or not did not confound the conclusions. However, in a study of chemotherapy-immune function-ICIs interaction, it was demonstrated that upfront multiline chemotherapy modulates the expression of antigen-presenting molecules, reduces bone marrow mobilization capacity, and interferes with the therapeutic response to subsequent ICIs [20]. Confusingly, studies have shown that chemotherapy may enhance the efficacy of ICIs by upregulating tumor-specific antigen expression [21, 22]. This disagreement may require more in-depth mechanistic studies and larger clinical trials.

To avoid missing risk factors, we further included in the multifactorial analysis of PFS and OS together with the variables identified in the univariate analysis with *P* < 0.1. In the multifactorial analysis, PS and OA treatment remained risk factors for the prognosis of ICIs treatment in lung cancer patients. Following adjustment for other confounding factors, the concomitant application of OA therapy remained an independent prognostic factor for PFS (HR = 4.994, 95% CI 3.217–7.753, *P* < 0.001) and OS (HR = 3.618, 95% CI 2.030–6.240, *P* < 0.001).

It must be acknowledged that there are still limitations in this study. First, as a retrospective cohort study, this study still has a small enrollment and population concentration in the same region, which may be biased, and the results of the subgroup analysis still need to be discussed and analyzed by a larger sample. Also, the inclusion of non-standard treatment patients improves comprehensiveness while limiting the generalization of findings across subgroups, and an expanded cohort may be helpful. In addition, since gut microbial composition is influenced by ethnic, dietary, and geographic differences, the impact of OA treatment in terms of resistance to ICIs may need to be confirmed in a different patient cohort [23, 24]. Second, the effect of OA on gut flora has been reported in some cancers, and its variation in lung cancer and its efficacy on ICIs remains to be further explored. Finally, because patients were not mandatorily tested for tumor PD-1/PD-L1 expression status prior to receiving ICIs, PD-1/PD-L1 status characteristics were not taken into account in the discussion, which may interfere with the comprehensiveness of the study.

It is not feasible to completely avoid OA treatment in clinical practice. Therefore, elucidating the detailed mechanism of OA affecting the efficacy of ICIs in lung cancer and finding appropriate methods to counteract the negative effect of OA on the efficacy of ICIs may be the direction of further research.

In conclusion, through retrospective analysis, this trial demonstrated that ICI is associated with diminished clinical outcomes in patients with OA. It provides a theoretical basis for subsequent extended cohort studies and prospective studies.

## Data Availability

The datasets generated and /or analysed during the current study are not publicly available, but are available from the corresponding author on reason- able request.
